# Immune Activation in HIV-Infected Aging Women on Antiretrovirals—Implications for Age-Associated Comorbidities: A Cross-Sectional Pilot Study

**DOI:** 10.1371/journal.pone.0063804

**Published:** 2013-05-28

**Authors:** Maria L. Alcaide, Anita Parmigiani, Suresh Pallikkuth, Margaret Roach, Riccardo Freguja, Marina Della Negra, Hector Bolivar, Margaret A. Fischl, Savita Pahwa

**Affiliations:** 1 Division of Infectious Diseases, University of Miami Miller School of Medicine, Miami, Florida, United States of America; 2 Department of Microbiology and Immunology, University of Miami Miller School of Medicine, Miami, Florida, United States of America; 3 Department of Surgery, Oncology and Gastroenterology, Section of Oncology and Immunology, Unit of Viral Oncology and AIDS Reference Centre, University of Padua, Italy; 4 Instituto de Infectologia Emílio Ribas, São Paulo, Brazil; 5 AIDS Clinical Research Unit, Division of Infectious Diseases, University of Miami Miller School of Medicine, Miami, Florida, United States of America; Rush University, United States of America

## Abstract

**Background:**

Persistent immune activation and microbial translocation associated with HIV infection likely place HIV-infected aging women at high risk of developing chronic age-related diseases. We investigated immune activation and microbial translocation in HIV-infected aging women in the post-menopausal ages.

**Methods:**

Twenty-seven post-menopausal women with HIV infection receiving antiretroviral treatment with documented viral suppression and 15 HIV-negative age-matched controls were enrolled. Levels of immune activation markers (T cell immune phenotype, sCD25, sCD14, sCD163), microbial translocation (LPS) and biomarkers of cardiovascular disease and impaired cognitive function (sVCAM-1, sICAM-1 and CXCL10) were evaluated.

**Results:**

T cell activation and exhaustion, monocyte/macrophage activation, and microbial translocation were significantly higher in HIV-infected women when compared to uninfected controls. Microbial translocation correlated with T cell and monocyte/macrophage activation. Biomarkers of cardiovascular disease and impaired cognition were elevated in women with HIV infection and correlated with immune activation.

**Conclusions:**

HIV-infected antiretroviral-treated aging women who achieved viral suppression are in a generalized status of immune activation and therefore are at an increased risk of age-associated end-organ diseases compared to uninfected age-matched controls.

## Introduction

With the advent of potent combination antiretroviral therapy (ART), improved survival benefits for persons with HIV infection have been well documented [1]. Despite the steady increase in longevity, the lifespan of HIV-infected persons still falls short of the average population, and they prematurely develop non-AIDS comorbidities such as cardiovascular disease (CVD), neurocognitive impairment, diabetes mellitus, osteoporosis and malignancies (reviewed in [2]).

The underlying bases for the development of end-organ diseases in the elderlies are not well understood, and are associated with a low-grade pro-inflammatory status termed inflammaging [3], attributed to immune dysregulation and senescence [4], [5]. In HIV infection as well, dysregulation of the immune system, characterized by an elevated status of immune activation (IA) and senescence, is considered to be a major contributing factor in disease progression [6], [7]. With the use of ART, IA decreases, but varying degrees of chronic immune activation persist even in virologically suppressed ART-treated HIV-infected persons [8]–[10]. Thus, the premature immune dysfunction in HIV infection resembles that of physiologic aging, and is the common thread underlying the non-AIDS metabolic conditions associated with aging and HIV infection. Menopause aggravates the aging process in women, and post-menopausal women are at a greater risk than men for these complications since the loss of sex hormones contributes to immune dysregulation [11] and senescence [12].

The mechanisms that lead to excessive IA in HIV infection are not fully determined. One major factor for IA is the translocation of microbial products in the bloodstream as a consequence of HIV-related damage of the intestinal mucosa [13], (reviewed in [14]. This microbial translocation (MT) is quantified by measuring plasma levels of lipopolysaccharide (LPS), a component of the cell wall of Gram-negative bacteria, (reviewed in [14]), [15]. A consequence of elevated LPS is activation of monocytes and macrophages and increased shedding of surface molecules such as soluble CD14 (sCD14) and CD163 (sCD163) [16], [17]. High LPS levels are also associated with phenotypic markers of T cell activation (CD38, HLA-DR) [15], implying a generalized state of activation that affects both the innate and adaptive arms of the immune system.

Many markers of IA have been correlated with poor clinical outcomes in HIV-infected patients. Among them, surface markers of T cell activation (CD38, HLA-DR) and senescence (loss of CD28, increased CD57 expression) are associated with subclinical carotid disease [18]. LPS and sCD14 correlate with poor CD4 T cell immune reconstitution; and sCD14 is linked to mortality and impaired cognitive function [9], [19]–[23]. Soluble CD163 is a predictor of non-calcified coronary plaques [24]; and soluble CD25 (sCD25), a subunit of interleukin 2 receptor on activated T cells, is associated with increased carotid intima media thickness [25]. Biomarkers of end-organ disease noted elevated in HIV infection include the soluble vascular cell adhesion molecule-1 (sVCAM-1) and soluble intracellular adhesion molecule-1 (sICAM-1) as novel biomarkers of CVD [26], and the chemokine CXCL10, indicative of impaired cognitive function [27], [28].

In this study, we hypothesized that, despites virological suppression, the combination of aging and HIV infection leads to chronic IA, thereby placing older HIV-infected women at higher risk of chronic diseases associated with aging in comparison to HIV-uninfected age-matched controls.

## Materials and Methods

### Ethics Statement

The study was approved by the University of Miami Institutional Review Board. Voluntary signed informed consent was obtained from every participant prior to participating in the study.

### Study Population

HIV-infected (HIV+) post-menopausal women who had plasma HIV RNA levels <100 copies/ml for at least six months while on ART, and a group of HIV-uninfected (HIV–) post-menopausal women as controls were enrolled. Women were referred to the study from local HIV providers and community clinics as well as HIV testing centers. Since menopause marks the aging process in women, and in order to avoid perimenopausal hormonal changes that may alter IA, we selected women that were in the post-menopausal state as defined by 12 months of amenorrhea. Women were considered eligible if they were older than 45 years of age and had not had a menstrual period in the prior 12 months. Women receiving hormonal replacement therapy, steroids, immunosuppressant medications, or with active malignancies were also excluded. HIV infection was documented by a positive licensed ELISA or EIA kit and confirmed by Western blot. Women enrolled in the control group had negative HIV test (rapid test or ELISA) prior to enrollment. The study was conducted at the University of Miami Center for AIDS Research (CFAR).

### Processing of Blood Samples

Blood was drawn by venipuncture and collected into heparinized tubes for plasma and peripheral blood mononuclear cells (PBMC) isolation. Samples were processed immediately after collection. Plasma was aliquoted and stored at −80°C until ready to be assayed. PBMC were isolated by standard Ficoll-hypaque density centrifugation, cryopreserved in FBS +10% DMSO, and stored in liquid nitrogen.

### Plasma Assays

#### sCD14 assay

Plasma levels of sCD14 were quantified by Human sCD14 Immunoassay (R&D Systems, Minneapolis, MN) following manufacturer’s instructions. Plasma was diluted 400-fold. Results were expressed in ng/ml.

#### sCD25, sCD163, sVCAM-1, sICAM-1 and CXCL10 assays

Plasma levels of these molecules were determined by the use of the specific DuoSet kits from R&D Systems, according to manufacturer’s instructions. For sCD25 measurement, samples were diluted 1∶2 and results were expressed in pg/ml. For sCD163 measurement, samples were diluted 1∶400 and results were expressed in ng/ml. For sVCAM-1 and sICAM-1, plasma samples were diluted 1,600 fold and analyte levels were expressed in ng/ml. For CXCl10 evaluation, samples were tested undiluted and diluted 1∶4; results were expressed in pg/ml.

#### LPS measurement

LPS levels were measured in plasma samples by the use of the Limulus amebocyte lysate chromogenic endpoint assay (Lonza Group Ltd, Allendale, NJ) according to the manufacturer’s recommendations. Samples were diluted 1∶5 in endotoxin-free water and heat-inactivated at 85°C for 10 minutes prior to the assay. LPS concentration in the samples was calculated in relation to an *E. Coli* endotoxin standard and expressed in pg/ml.

#### Multiplex cytokine measurement

Plasma levels of cytokines were measured using a customized MILLIPLEX™ Cytokine Human Ultrasensitive magnetic bead panel (EMD Millipore, Billerica, MA) following manufacturer’s instructions. Briefly, plasma samples were thawed, vortexed and centrifuged at 10,000 rpm for 5 min at 4°C immediately prior to testing. Undiluted plasma was incubated over night with a mixture of beads specific for interleukin(IL)-6, IL-8, IL-10 and tumor necrosis factor alpha (TNFα) at 4°C with shaking. After washing, the beads were incubated with biotinylated detection antibodies for 1 hour at room temperature. Streptavidin-PE was then added to the wells and allowed to incubate for 30 minutes at room temperature. The beads were then washed and diluted with 150 µl Sheath Fluid before acquisition on a MAGPIX instrument (Luminex Corporation, Austin, TX). The mean fluorescent intensity (MFI) data were analyzed with MILLIPLEX™ Analyst Software V.3.5 (EMD Millipore). Cytokine concentrations were determined based on standard curves and expressed in pg/ml.

### Multicolor Flow Cytometry

Monoclonal antibodies (MoAbs) utilized for PBMC staining were: CD3 AmCyan, CD28 PE-Cy7, CD57 FITC, Ki-67 PerCP-Cy5.5 and HLA-DR AF700 from BD Biosciences (San Jose, CA); CD8 QDot605, CD4 QDot655 and CD38 PE Texas Red from Invitrogen (Eugene, OR); PD-1 PE from eBioscience (San Diego, CA); CD127 PE-Cy5 from Beckman Coulter (Indianapolis, IN). Live/Dead® Fixable Violet Dead Cell Stain (ViViD) Kit (Invitrogen) was included in all staining panels for exclusion of dead cells. Appropriate isotype control MoAbs were used for proper gating. Frozen PBMC were thawed, rested overnight in complete medium (RPMI 1640 supplemented with 10% FBS) and counted. One million cells were incubated with ViViD stain and MoAbs for surface markers for 15 minutes in the dark. After incubation, cells were washed, fixed, permeabilized with Cytofix/Cytoperm Buffer (BD Biosciences), and finally stained for intracellular marker Ki-67 for 30 minutes in the dark. Cells were then washed and resuspended in PBS +1% paraformaldehyde. Samples were acquired on the BD Biosciences LSRFortessa analyzer after proper instrument setting and compensation [29], [30]. At least 500,000 events in the lymphocyte gate were acquired per sample. Data analysis was performed using the FlowJo software version 8.8.6 (TreeStar, San Carlos, CA). Frequencies of desired subsets were determined in gated live (ViViD negative) cells.

### Statistical Analysis

Differences between groups were analyzed by Student *t*-test, the 2-sample Wilcoxon rank-sum (Mann-Whitney) test, or Fisher’s exact test, according to data distribution. Correlations between two variables were evaluated by Pearson correlation and linear regression. Analyses were performed using GraphPad Prism 5 (GraphPad Software Inc, La Jolla, CA). P values <0.05 were considered significant.

## Results

### Characteristics of Study Population

Twenty-seven HIV+ women were enrolled in the study. All women were receiving antiretroviral therapy accordingly to the standard of care and had received ART for more than 6 months. ART included two nucleoside reverse transcriptase inhibitors with either a Ritonavir boosted protease inhibitor, the non-nucleoside reverse transcriptase inhibitor Efavirenz or the integrase inhibitor Raltegravir. Characteristics of the study population are summarized in **Table 1**. Plasma HIV-RNA was <100 copies/ml in all patients. The majority of patients (67%) had >500 CD4 cells/mm^3^ and 75% had a nadir CD4<200 cells/mm^3^. Fifteen HIV– women were enrolled as a control group. Since the loss of menstrual period is a sign of aging in women, HIV+ and HIV– women were matched for age and time to menopause. Rates of illicit drug use and smoking were higher (although not significantly) in the HIV– group, likely due to the low income area were the referring clinics were located (**Table 1**).

**Table 1:** Characteristics of the study population.

	HIV– women	HIV+ women	P value
	n = 15	n = 27	
**Age** (years)	59 (53–63)	56.5 (48–66)	0.11
**Time to menopause** (years)	15 (3–29)	12 (2–22)	0.09
**CD4 cell count** (cells/mm^3^)	n.a.	584 (144–1,144)	
**CD4 nadir** (cells/mm^3^)	n.a.	147 (2–648)*	
**HIV RNA** (copies/ml)	n.a.	undetectable–80	
**Current smoking**	13%	8%	0.61
**Current illicit drug use**	6%	4%	1.00
**Body mass index** (kg/m^2^)	31.4 (21.5–38.1)	27.6 (20.1–38.3)	0.20

* n = 25.

Menopause was defined as lack of menstruation for more than 12 months. Lower limit for plasma HIV RNA detection was 20 copies/ml. Body mass index was calculated as the weight in kilograms divided by the square of the height in meters. P values were calculated using Mann-Whitney, Student *t*-test or Fisher’s exact test as appropriate.

### T Cell Activation and Senescence are Increased in HIV+ Post-menopausal Women

In adults with chronic HIV infection, markers of T cell activation are reported to be elevated even after antiretroviral therapy [8]. We evaluated cell phenotype markers of T cell activation (CD38, HLA-DR, Ki-67) and exhaustion (PD-1) in our study population. As shown in **Table 2**, surface expression of CD38 and HLA-DR in CD4 and CD8 T cells was elevated in the HIV+ group as compared to the HIV– controls. In the HIV+ women, CD4, but not CD8 T cells, displayed significantly higher levels of Ki-67 and PD-1.

**Table 2:** Cellular markers of T cell activation, exhaustion and senescence in HIV– and HIV+ post-menopausal women.

	HIV– women	HIV+ women	P value
	n = 13	n = 22	
**T cell activation**			
CD38+ HLA-DR+ CD4 (%)	1.69±0.95	3.21±1.87	**0.0313**
CD38+ HLA-DR+ CD8 (%)	2.08±1.39	10.17±13.26	**<0.0001**
Ki-67+ CD4 (%)	0.39±0.22	0.63±0.29	**0.0260**
Ki-67+ CD8 (%)	0.32±0.09	0.34±0.18	0.6913
**T cell exhaustion**			
PD-1+ CD4 (%)	13.36±6.81	21.99±11.80	**0.0321**
PD-1+ CD8 (%)	16.72±9.86	20.50±7.34	0.2177
**T cell senescence**			
CD28– CD57+ CD4 (%)	2.22±2.61	9.43±12.24	**0.0390**
CD28– CD57+ CD8 (%)	16.07±10.40	24.59±13.88	**0.0481**
CD127 CD4 (MFI)	3,457±901	2,737±890	**0.0368**
CD127 CD8 (MFI)	1,795±850	1,093±930	0.0512

Expression of activation (CD38, HLA-DR, Ki-67), exhaustion (PD-1) and senescence (CD28, CD57, CD127) markers was evaluated by flow cytometry in live CD4 and CD8 T cells. Cryopreserved PBMC were thawed and rested overnight before staining with ViViD and monoclonal antibodies and subsequent acquisition on a flow cytometer. Gating strategy for the phenotypic analysis of T cells was performed as follows: Lymphocytes were gated based on forward and side scatter, and gates for exclusion of singlets and dead cells (ViViD+ events) were drawn. Statistical differences between groups were analyzed by Student *t*-test. Significant P values are shown in bold.

Accelerated T cell senescence, phenotypically described as loss of the costimulatory molecule CD28 and acquisition of the exhaustion marker CD57, has been associated with HIV infection [31]. In our study cohort, we observed increased levels of senescent T cells in the HIV+ group as compared to the HIV– controls (**Table 2**). Surface expression of CD127 (IL-7 receptor α chain) is a feature of long-living memory T cells, and its down-modulation is associated with loss of CD4 T cells in HIV infection [32]. Analysis of CD127 expression in CD4 and CD8 T cells revealed a decrease in this marker in the HIV+ women, and it was more pronounced in the CD4 T cell compartment (**Table 2**).

### Soluble Markers of Immune Activation and Microbial Translocation are Increased in HIV+ Post-menopausal Women

In order to assess the level of activation in monocytes and macrophages of aging women, sCD14 and sCD163 were evaluated. Levels of sCD14 and sCD163 were significantly higher in the plasma of HIV+ group of women when compared to the uninfected women (**Table 3**). Similarly, sCD25, that is shed by activated T cells, was also increased in the HIV+ women as compared to the HIV– controls (**Table 3**).

**Table 3:** Soluble markers of immune activation and microbial translocation in HIV– and HIV+ post-menopausal women.

	HIV– women	HIV+ women	P value
	n = 15	n = 27	
**Monocyte/macrophage activation**			
sCD14 (ng/ml)	1,537±253	2,113±426	**<0.0001**
sCD163 (ng/ml)	323±155	533±260	**0.0043**
**T cell activation**			
sCD25 (ng/ml)	387.3±151.2	590.1±425.6	**0.0423**
**Cytokines**			
IL-6 (pg/ml)	0.89±0.17	1.86±0.44	0.0728
IL-8 (pg/ml)	4.38±0.52	6.57±1.26	0.1012
IL-10 (pg/ml)	3.31±1.58	19.74±4.85	**0.0124**
TNFα (pg/ml)	7.02±1.43	9.58±1.23	0.1359
**Microbial translocation**			
LPS (pg/ml)	90.2±21.4	107.4±20.7	**0.0221**

Circulating levels of sCD14, sCD163 and sCD25 were measured in the plasma of 27 HIV+ post- women and 15 HIV– controls by ELISA. Plasma levels of cytokines were measured using a customized MILLIPLEX™ Cytokine Human Ultrasensitive magnetic bead panel (EMD Millipore). LPS levels were measured in plasma samples by the use of the Limulus amebocyte lysate chromogenic endpoint assay, as described in the Methods. Statistical differences between groups were analyzed by Student *t*-test. P values <0.05 are shown in bold.

Since activated monocytes and T cells are prone to produce inflammatory cytokines, we evaluated the plasma levels of pro-inflammatory mediators. Although differences between HIV+ and HIV– subjects were not significant, increased levels of circulating IL-6, IL-8 and TNFα were observed in the HIV+ group (**Table 3**). Plasma IL-10, highly produced by T, B, and natural killer cells of HIV+ subjects [33], was also elevated in the HIV+ women as compared to the HIV– controls (**Table 3**).

Circulating LPS is an indicator of translocation of microbial products into the bloodstream. Chronically HIV+ women had significantly higher levels of plasma LPS than uninfected controls (**Table 3**).

### Biomarkers of Cardiovascular Disease and Impaired Cognition are Elevated in HIV+ Post-menopausal Women

Soluble VCAM-1 and ICAM-1, adhesion molecules shed by activated endothelial cells, are considered biomarkers of cardiovascular disease [26]. Both sVCAM-1 and sICAM-1 were elevated in HIV+ women (**Table 4**).

**Table 4:** Biomarkers of cardiovascular disease and impaired neurocognition are increased in HIV-infected post-menopausal women.

	HIV– women	HIV+ women	P value
	n = 15	n = 26	
**Cardiovascular disease**			
sVCAM-1 (ng/ml)	287.0±71.3	397.8±136.0	**0.0073**
sICAM-1 (ng/ml)	100.4±28.1	171.5±82.9	**0.0037**
**Impaired neurocognition**			
CXCL10 (pg/ml)	338.9±235.3	849.7±608.9	**0.0035**

Levels of sVCAM-1, sICAM-1 and CXCL10 were measured by ELISA in the plasma of 15 HIV– and 26 HIV+ women. Comparisons between the two groups were performed using Student *t*-test. Significant P values are shown in bold.

CXCL10 is found to be increased in HIV+ patients with HIV-associated neurologic disorders [34], wherein it is considered to be neurotoxic. Levels of this chemokine were higher in the HIV+ women than in the HIV– controls (**Table 4**).

### High Microbial Translocation and Low CD4 Count in HIV+ Post-menopausal Women are Associated with Increased Immune Activation

**Figure 1:**
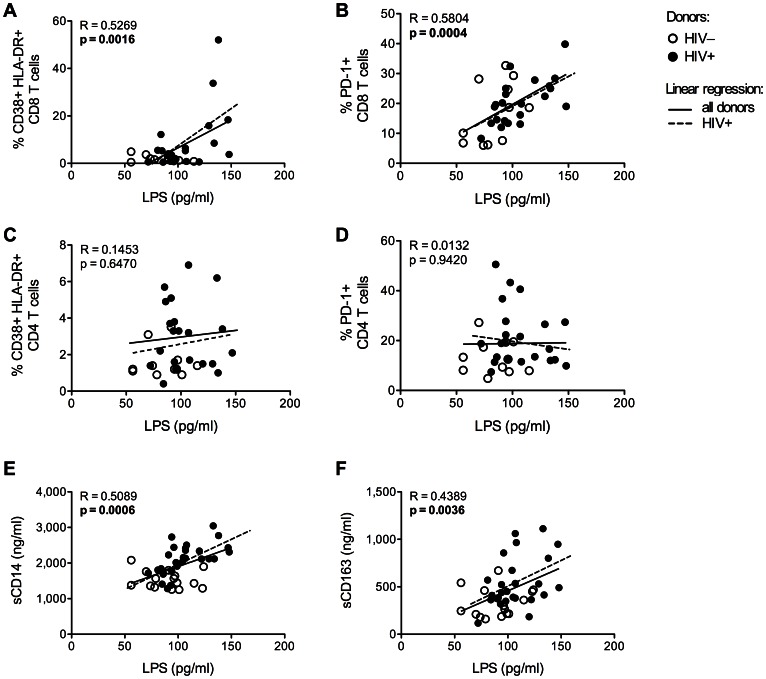
Elevated levels of microbial products in the bloodstream of HIV+ post-menopausal women are associated with the extent of immune activation. Plasma levels of LPS were measured by the use of the Limulus amebocyte lysate chromogenic endpoint assay. Expression of activation (CD38, HLA-DR) and exhaustion (PD-1) markers was evaluated by flow cytometry in live CD4 and CD8 T cells. Circulating levels of sCD14 and sCD163 were measured by ELISA. (**A–D**) Graphs show the correlation between plasma LPS levels and: frequency of activated (**A**) and exhausted (**B**) CD8 T cells, and of activated (**C**) and exhausted (**D**) CD4 T cells. Correlations were established for 11 HIV– and 22 HIV+ women (continuous lines), and for the HIV+ women alone (dashed lines). (**E, F**) Graphs depict the correlation between plasma levels of LPS and those of sCD14 (**E**) or sCD163 (**F**) of 42 women (15 HIV–, open dots, and 27 HIV+, filled dots). Correlation between the two variables is indicated by the continuous line. Dashed line shows the correlation between the two variables when only the HIV+ subjects were taken into account. Significant P values are shown in bold.

Persistent exposure to microbial products has been suggested as an important mechanism driving IA in adult HIV patients [15], [35], but the interplay between microbial translocation and immune activation in aging women is unclear. We observed that the extent of CD8 T cell activation (%CD38+ HLA-DR+ CD8 T cells) and exhaustion (%PD-1+ CD8 T cells) was strongly associated with the plasma levels of LPS (**Fig. 1** **A** and **B**). However, activation and exhaustion of CD4 T cells were not associated with the extent of MT (**Fig. 1** **C** and **D**), neither was CD4 and CD8 T cell senescence (not shown). Moreover, levels of circulating LPS were associated with monocyte activation (sCD14 and sCD163) (**Fig. 1** **E** and **F**, continuous line). Of note, the correlation between MT and monocyte activation was more pronounced when the analysis was restricted to HIV+ subjects (**Fig. 1** **E** and **F**, dashed line).

**Figure 2:**
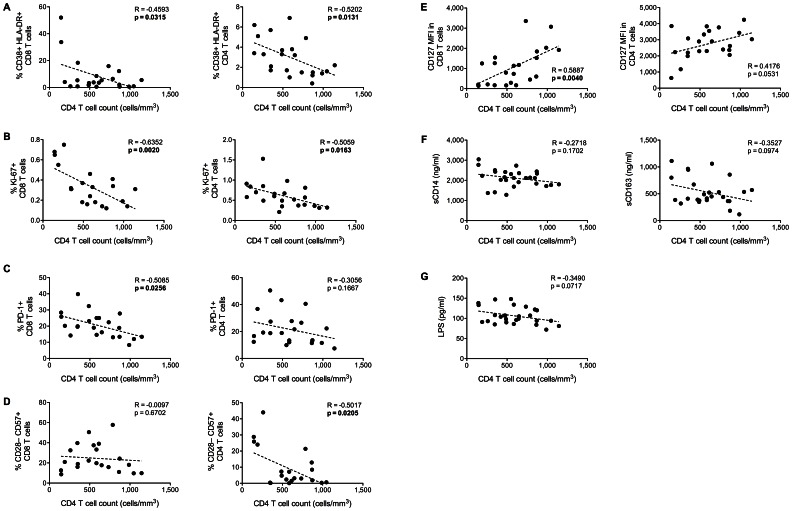
Association between CD4 count, IA and MT in HIV+ post-menopausal women. Markers of T cell activation (CD38, HLA-DR), exhaustion (PD-1) and senescence (loss of CD127 and CD28, CD57 expression) were established in live CD8 and CD4 T cells by multicolor flow cytometry. Circulating levels of sCD14 and sCD163 were measured by ELISA. Plasma LPS levels were measured by the Limulus amebocyte lysate chromogenic endpoint assay. (**A–E**) Graphs show correlation between CD4 T cell count and markers of T cell activation (**A, B**), exhaustion (**C**) and senescence (**D, E**) for 22 HIV+ donors. (**F**) Correlation between CD4 count and monocyte/macrophage activation markers sCD14 and sCD163 for 27 HIV+ women. (**G**) Correlation between CD4 count and plasma levels of LPS for 27 HIV+ women. Significant P values are shown in bold.

Several groups have shown that in young HIV+ adults with viral suppression on ART, failure to normalize CD4 T cell count is related to immune activation [36]–[38]. We observed inverse association between CD4 cell count and the frequency of activated- (**Fig. 2** **A**), dividing- (**Fig. 2** **B**), and exhausted (**Fig. 2** **C**) T cells. Low CD4 count was also associated with accumulation of senescent CD4 (but not CD8) T cells (**Fig. 2** **D**), and with low expression of CD127 (**Fig. 2** **E**).

We also observed a modest inverse association between CD4 count and monocyte/macrophage activation (**Fig. 2** **F**), overall indicating that in HIV+ women who are ART-treated and virally suppressed the extent of immune activation negatively correlates with CD4 cell count.

Since MT is an important factor in promoting IA, we investigated whether it would have any association with CD4 cell count. As shown in **Fig. 2** **G**, the extent of microbial translocation in the HIV+ group was inversely associated with CD4 cell count, suggesting that preservation of the CD4 T cell subset may contribute to limit the HIV-induced damage to the gut, thus containing the translocation of microbial products to the bloodstream, and the subsequent IA.

### Increased Levels of Biomarkers of CVD and Impaired Cognitive Function are Associated with High Immune Activation and Low CD4 Count

**Figure 3:**
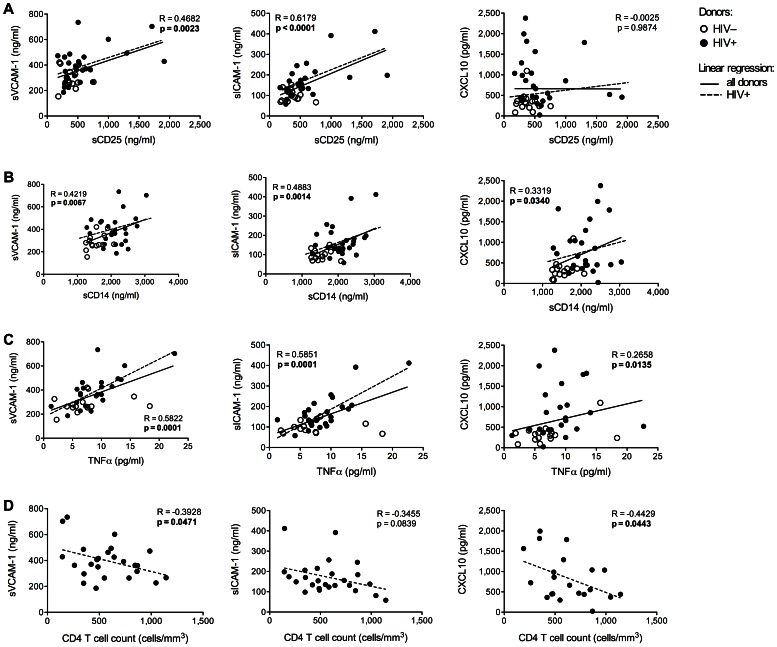
Biomarkers of CVD and cognitive impairment correlate with the status of immune activation and CD4 T cell count. Correlation between the circulating levels of sVCAM-1, sICAM-1 and CXCL10 and those of plasma sCD25 (**A**), sCD14 (**B**), TNFα (**C**) and CD4 cell count (**D**). In each graph, the continuous line indicates the correlation between the two variables for all donors (HIV– and HIV+), while the dashed line shows the correlation between the two variables when only the HIV+ donors were taken into account. P values <0.05 are shown in bold.

In order to establish potential outcomes of abnormal IA, MT and CD4 cell count observed in the HIV+ women, we sought to determine whether they would correlate with biomarkers of cardiovascular disease (sVCAM-1, sICAM-1) and impaired cognitive function (CXCL10). We observed a direct association between these biomarkers and the levels of T cell (**Fig. 3** **A**) and monocyte/macrophage (**Fig. 3** **B**) activation, as well as with the levels of the pro-inflammatory cytokine TNFα (**Fig. 3** **C**). Circulating levels of sVCAM-1, sICAM-1 and CXCL10 inversely correlated with CD4 T cell count (**Fig. 3** **D**). No association was observed with LPS levels (not shown).

## Discussion

Persistent immune activation and inflammation associated with HIV infection accelerate the process of immunosenescence and systemic aging, likely placing HIV+ aging women at higher risk of developing chronic age-related diseases such as CVD and impaired cognitive function. We demonstrate that aging HIV+ women manifest higher state of immune activation and increased levels of biomarkers associated with CVD and impaired neurocognition than HIV^–^ age-matched controls. Increased levels of sCD14 and sCD163 were indicative of monocyte/macrophage activation; and T cell activation was implied by elevated plasma sCD25, increased expression of CD38, HLA-DR, and intracellular Ki-67. T cell activation was accompanied by increase in PD-1 and CD57 that are designated markers of exhaustion and senescence respectively. We also found increased levels of microbial translocation that correlated with markers of IA and T cell exhaustion. These data represent the first comprehensive analysis of the effect of HIV infection on the status of the immune system in aging women who are post-menopausal and are on ART with controlled viremia. These findings are significant because they occur in an age group where the combination of inflammaging (the upregulation of inflammatory markers that normally occurs in the elderlies) and loss of sex hormones are likely to increase the risk of age-related comorbidities.

Among factors implicated for triggering IA in HIV-infected patients, microbial translocation is one of the most prominent [9], [15], [29], reviewed in [31]. The presence of bacteria-derived products in the bloodstream [15], [35] is sensed by toll-like receptors [39], that are responsible for inducing the production of inflammatory mediators that contribute to systemic immune activation. MT has been associated with severity of HIV infection [28], [29], [40], and elevated levels of LPS in the plasma of HIV+ ART-treated individuals have been used as an indicator of long-lasting damage to the gut [35], reviewed in [31]. In our study, we observed increased plasma levels of LPS in HIV+ aging women when compared to the uninfected controls. In the HIV+ cohort, a significant association was noted between LPS levels and the extent of monocyte/macrophage and CD8 T cell activation and exhaustion. These results suggest the MT was still occurring in HIV+ aging women despite plasma viral suppression and was contributing to persistent IA.

A second cause of IA is CD4 T cell depletion. Lymphopenia perturbs the physiological cytokine network [41], stimulating T cell homeostatic proliferation and IA [42]. Failure of immune recovery despite the use of ART in younger adults is associated with IA [36]–[38]. We found a significant correlation of low CD4 counts with higher rates of T cell activation (CD38, HLA-DR), proliferation (Ki-67) and exhaustion (PD-1) in CD8 and CD4 T cells, indicating that T cell dysfunction and immune senescence are associated with low CD4 counts. Although not significant, LPS levels also had an inverse relationship with CD4 count. We have recently shown in rhesus macaques that SIV-mediated CD4 T cell depletion in the gut was controlled by the administration of exogenous IL-21 and resulted in lower levels of plasma LPS compared to untreated animals, who had a more severe CD4 T cell depletion (SP, manuscript submitted). Collectively, these findings suggest that early ART initiation in HIV infection before depletion of CD4 cells may be of great importance in aging women to limit MT and IA.

HIV viremia has been associated with increased T cell activation and proliferation [43], [44]. In order to reduce variability in IA status due to different levels of viral load, HIV+ women with plasma virus levels of <100 copies/ml were enrolled in our study. However, some degree of IA has been attributed to low-level viral replication that can occur in ART-treated patients with plasma viral RNA levels below the limit of detection [45], [46]. The extent of direct HIV-induced IA in such patients is controversial and difficult to quantitate, and we could not rule out its effect in our study group.

The importance of systemic immune activation and senescence in patients with HIV infection is reflected in the observation that several markers of IA, found to be elevated in our cohort of HIV+ women, are associated with important clinical outcomes. Soluble CD14, sCD25 and sCD163, as well as expression of CD38, HL-DR, CD57 and loss of CD28 have been associated with subclinical CVD and decreased cognitive function in HIV infection [2], [7], [18], [20], [24], [25]. Furthermore, we found that biomarkers of CVD (sVCAM-1, sICAM-1) and of impaired neurocognition (CXCL10) were higher in women with HIV infection, and strongly correlated with the state of IA. Other important risk factors for CVD such as smoking [47] and BMI [48] were comparable between the two groups and would therefore not confound the study results. In this pilot study we did not perform clinical tests to determine evidence of CVD or neurocognitive function. Imaging studies performed in men and women with controlled and uncontrolled HIV infection have documented the association between cardiovascular risk and IA markers [18], [24], [25]. Albeit our results describe associations and do not prove causality, they support the hypothesis that women with well-controlled HIV infection on ART have an increased risk of developing CVD and neurocognitive disorders compared to age-matched uninfected women, and that the underlying basis is the IA and inflammation reflecting collateral damage imposed by HIV.

Although other studies have evaluated IA and MT in HIV infection, this study is unique since it focuses on aging women who are post-menopausal, providing a specific criterion for aging and gender. Elderly women constitute a growing group of women at higher risk of developing age-related complications due to the combination of aging, HIV infection and loss of ovarian function. Few studies have evaluated the difference on IA in aging individuals by gender and it has recently been suggested that markers of IA (sCD14, sCD163 and CXCL10) are elevated in aging women when compared to men [49]. We speculate that aging women have higher levels of IA markers than men and this may be due, at least in part, to the loss of sex hormones occurring after menopause.

Limitations of the study include the small sample size, absence of younger women and older men for comparison, and absence of sexual hormones levels. As a pilot, however, this study demonstrates that HIV-infected antiretroviral-treated aging women who achieved viral suppression have higher levels of immune activation, exhaustion and senescence than HIV-uninfected age-matched controls; and that IA is associated with biomarkers of CVD and neurocognitive deterioration. Further studies are needed to identify the IA components that correlate with different clinical outcomes in HIV-infected women in this age group. Other causes of systemic IA such as concurrent chronic infections (i.e. EBV, CMV, HCV), HIV viral reservoirs and residual viral replication will help to understand the mechanisms driving IA in HIV controlled infection. As recently suggested, viral persistence is facilitated by IA [50], and strategies to contain IA including control of MT need to be explored in this patient group.
